# Degradation of misfolded proteins in neurodegenerative diseases: therapeutic targets and strategies

**DOI:** 10.1038/emm.2014.117

**Published:** 2015-03-13

**Authors:** Aaron Ciechanover, Yong Tae Kwon

**Affiliations:** 1 Protein Metabolism Medical Research Center and Department of Biomedical Sciences, College of Medicine, Seoul National University, Seoul, Korea; 2 Tumor and Vascular Biology Research Center, The Rappaport Faculty of Medicine and Research Institute, Technion-Israel Institute of Technology, Haifa, Israel

## Abstract

Mammalian cells remove misfolded proteins using various proteolytic systems, including the ubiquitin (Ub)-proteasome system (UPS), chaperone mediated autophagy (CMA) and macroautophagy. The majority of misfolded proteins are degraded by the UPS, in which Ub-conjugated substrates are deubiquitinated, unfolded and cleaved into small peptides when passing through the narrow chamber of the proteasome. The substrates that expose a specific degradation signal, the KFERQ sequence motif, can be delivered to and degraded in lysosomes via the CMA. Aggregation-prone substrates resistant to both the UPS and the CMA can be degraded by macroautophagy, in which cargoes are segregated into autophagosomes before degradation by lysosomal hydrolases. Although most misfolded and aggregated proteins in the human proteome can be degraded by cellular protein quality control, some native and mutant proteins prone to aggregation into *β*-sheet-enriched oligomers are resistant to all known proteolytic pathways and can thus grow into inclusion bodies or extracellular plaques. The accumulation of protease-resistant misfolded and aggregated proteins is a common mechanism underlying protein misfolding disorders, including neurodegenerative diseases such as Huntington's disease (HD), Alzheimer's disease (AD), Parkinson's disease (PD), prion diseases and Amyotrophic Lateral Sclerosis (ALS). In this review, we provide an overview of the proteolytic pathways in neurons, with an emphasis on the UPS, CMA and macroautophagy, and discuss the role of protein quality control in the degradation of pathogenic proteins in neurodegenerative diseases. Additionally, we examine existing putative therapeutic strategies to efficiently remove cytotoxic proteins from degenerating neurons.

## Introduction

**Figure 1:**
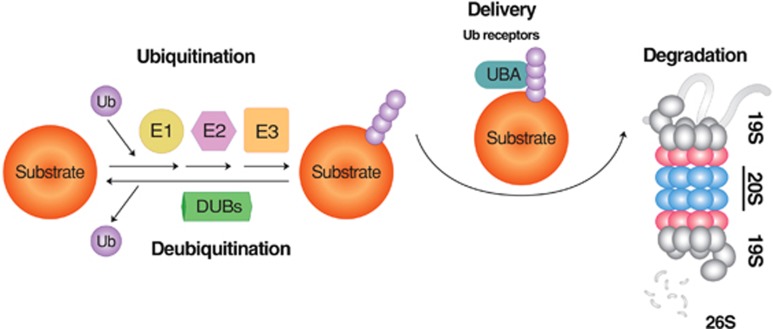
The degradation of short-lived proteins by the UPS. In this selective proteolytic system, Ub is first activated by E1 and subsequently transferred to E2. In parallel, misfolded substrates of the UPS are recognized by molecular chaperones, such as CHIP, and associated with Ub ligases that promote the transfer of E2-conjugated Ub to specific Lys residues of substrates. Ubiquitinated substrates are deubiquitinated, unfolded, fed into the narrow chamber of the proteasome, and progressively cleaved into small peptides. Depending on the types of E3 ligases, Ub can be directly transferred from E2 to the substrate or via a two-step process that involves a transient binding of E3 to Ub. The repetition of this reaction results in the growth of a singly conjugated Ub to a chain of Ub with different topologies, depending on how Ub is conjugated to another Ub. Modified from Wang and Robbins.^226^

**Figure 2:**
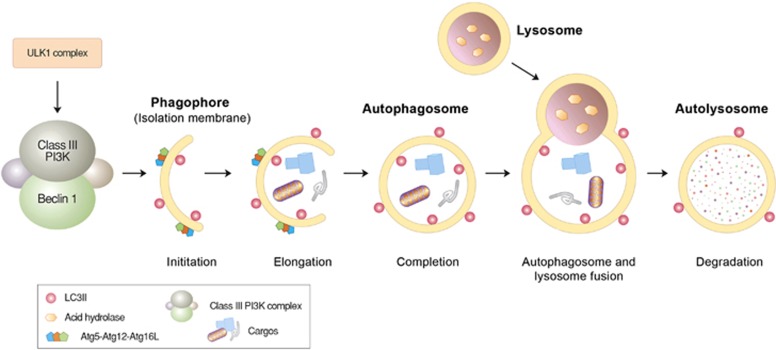
Autophagosome formation and lysosomal degradation. Autophagosome formation can be triggered when the mTOR complex is inhibited by various stressors, such as starvation. This induces the assembly of the ULK protein complex composed of ULK1, Atg13 and FIP200 at the isolation membrane, which, in turn, activates the formation of the Beclin-1/PI3KC3 complex composed of Beclin-1, UVRAG, Bif-1, Ambra1, Vps15 and Vps34. During the elongation of the isolation membrane, the Atg5-Atg12-Atg16L1 complex mediates the conjugation of PE to LC3-I, generating LC3-II that relocates from the cytosol to the autophagic membrane and is anchored on its surface. The resulting autophagic membrane structures—autophagosomes—are fused with lysosomes to form autolysosomes, wherein cargoes, including misfolded proteins, are degraded by lysosomal hydrolases.

**Figure 3:**
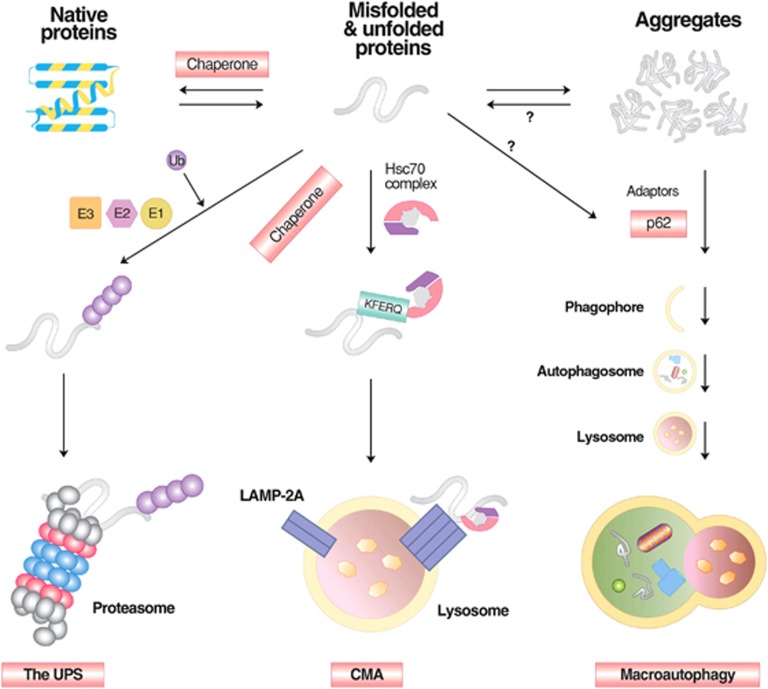
The degradation of misfolded proteins by various cellular proteolytic pathways. Misfolded proteins are initially recognized by molecular chaperones that deliver the substrates to the UPS, CMA or macroautophagy depending on the nature of misfolding, size and solubility. In general, soluble and monomeric misfolded proteins are primarily degraded by the UPS and CMA. In CMA, substrates carrying the KFERQ motif are recognized and bound by Hsc70 in association with chaperones. The substrates are subsequently delivered to the LAMP2 complex on the lysosomal membrane, translocated to the lumen, and degraded into amino acids by lysosomal hydrolases. Some of these misfolded proteins tend to form aggregates and are thus directed to macroautophagy. Misfolded protein substrates of macroautophagy are recognized by molecular chaperones such as Hsc70, ubiquitinated by Ub ligases, and delivered to the autophagic adaptor p62, leading to the formation of p62 protein bodies. The targeted protein aggregates associated with p62 are subsequently delivered to autophagic membranes for lysosomal degradation, when p62 interacts with LC3 on the autophagic membrane.

Misfolded proteins generated in various cellular compartments, including the cytoplasm, nucleus and endoplasmic reticulum (ER), are efficiently removed by quality control systems composed of the ubiquitin (Ub)-proteasome system (UPS), chaperone mediated autophagy (CMA) and macroautophagy (Figure 1).^1^ The first line of defense in degrading soluble misfolded proteins is the UPS (Figure 2), a selective proteolytic system in which substrates are tagged with Ub, unfolded into nascent polypeptide chains, and cleaved into short peptides while passing through the narrow chamber of the proteasome.^1, 2, 3, 4^ Specific misfolded proteins that expose the KFERQ degradation signal can be degraded by the CMA, a branch of the autophagy-lysosome system (hereafter autophagy), in which substrates are selectively recognized by the chaperone heat-shock cognate 70 (Hsc70) and directly delivered into lysosomes, leading to degradation by lysosomal hydrolases into amino acids (Figure 1).^5, 6^ Some misfolded proteins that escape the surveillance of the UPS and CMA or tend to form aggregates are directed to macroautophagy (Figure 1), a bulk degradation system in which substrates are segregated into autophagosomes which, in turn, are fused with lysosomes for degradation into amino acids (Figure 3).^7, 8^ Although almost all of the proteins encoded by the human genome can be efficiently removed from the cell when misfolded, a number of polypeptides generated from post-translational conjugation (for example, hyperphosphorylated tau in Alzheimer's disease (AD)) or endoproteolytic cleavage (for example, amyloid β peptides) tend to be spontaneously misfolded and rapidly aggregated into oligomers enriched in β-sheet content.^9, 10, 11, 12^ Genetic mutations in specific proteins, such as huntingtin in Huntington's disease (HD),^13, 14^ α-synuclein in Parkinson's disease (PD),^15, 16^ prion protein (PrP) in prion diseases,^17, 18, 19^ and superoxide dismutase 1 (SOD1) and TAR DNA-binding protein 43 kDa (TDP-43) in Amyotrophic Lateral Sclerosis (ALS),^20^ may also perturb their folding, leading to the formation of similar β-sheet-enriched aggregates. The resulting oligomers are at least partially resistant to all known proteolytic pathways and can further grow into inclusion bodies or extracellular plaques that have highly ordered fibrillar structures with elevated β-sheet content.^9^ Cytotoxicity and neuronal death caused by misfolded oligomers and aggregates provide a molecular mechanism underlying the pathogenesis of many neurodegenerative diseases.^21^

Compared with proliferating cells, post-mitotic neurons are more sensitive to the accumulation of cytotoxic proteins because they cannot dilute toxic substances by means of cell division.^22^ Moreover, protein quality control is intrinsically challenging in neurons because of their unique cellular structure, characterized by the expansion of dendrites and axons in which protein aggregates need to be packaged into autophagic vacuoles and make a retrograde journey to the cell body, rich in lysosomes, for degradation.^23, 24^ Although young neurons can manage to clear cytotoxic proteins, this task becomes increasingly more difficult throughout the course of aging during which the components of the UPS, CMA and macroautophagy are downregulated in expression and activity.^25, 26^ In the affected neurons of many neurodegenerative diseases, such as AD, PD, HD, prion diseases and ALS, pathogenic protein aggregates can further downregulate the activities of proteolytic pathways.^27, 28, 29, 30, 31, 32^ One way to enhance degradation of pathogenic protein aggregates is to increase the activities of proteolytic pathways. Many small molecule compounds have been developed and successfully used to enhance the clearance of various pathogenic proteins.^33, 34, 35, 36, 37, 38^

## The UPS in neurodegenerative diseases

The UPS is a proteolytic system in which the conjugation of Ub to substrates induces selective degradation by the proteasome (Figure 2).^39^ Protein degradation in the UPS is mediated by an enzymatic cascade composed of ~500–1000 proteins. In this ATP-consuming proteolytic system, Ub is first activated by forming a thioester bond between its C-terminal Gly76 residue and an active-site cysteine (Cys) of the Ub-activating enzyme E1. The activated Ub is transferred to the Ub-conjugating enzyme E2 via a thioester bond. It is the Ub ligase E3 that selectively recognizes and mediates ubiquitination of substrates, which involves the transfer of E2-conjugated Ub to lysine (Lys) residue(s) of the target substrate. The human genome is estimated to encode >500 E3 ligases, which can be classified into three groups depending on the types of ubiquitination domains, including the really interesting new gene (RING) finger, the homologous to E6-AP (HECT) domain and the U-box domain.^40^ An E3 Ub ligase can be a single polypeptide or a subunit of a protein complex, such as the SCF (Skp1-Cullin1-F-box) E3 complex. As Ub conjugation may occur at any of its seven Lys residues, a Ub chain can grow into many different topologies.^41^ The Lys48 linkage is the most widely used topology, which signals degradation by the proteasome, whereas the Lys63 linkage mediates non-proteolytic processes, such as Ub-dependent protein–protein interactions.^42^ The Lys11 linkage is typically used for cell-cycle regulation and cell division.^43^ Ub moieties on protein substrates can be removed by the deubiquitination enzyme to edit elongating chains or remove/recycle the targeted chains altogether from substrates.^44, 45^

Once ubiquitination generates a chain of four or more Ub at lysine 48, it can serve as a secondary degron that delivers the substrates to the 26S proteasome. This cylindrical machinery is composed of a proteolytic 20S core particle capped at both ends by a 19S regulatory particle.^46, 47, 48^ The 19S particle binds and unfolds the polyubiquitinated protein substrate and feeds the unfolded polypeptide chain into the chamber of the 20S particle, which is as narrow as 13 angstroms in diameter.^47, 48^ When feeding the substrates into the 20S particle, the 19S particle also deubiquitinates the polyubiquitinated substrates to recycle Ub. Passing through the 20S particle, the substrates are cleaved into small peptides by the β5, β2 and β1 subunits that have chymotrypsin-like, trypsin-like and caspase-like peptidase activities, respectively.^47, 48^

Substrates of the UPS include misfolded proteins, as well as a large number of short-lived proteins in the cytoplasm, nucleus, ER and other cellular compartments. The UPS-dependent degradation of misfolded proteins initiates when chaperones and Ub ligases recognize abnormalities in folding, such as hydrophobic residues exposed on the surface and improper disulfide bonds.^49^ Several E3s are known to mediate the ubiquitination of misfolded proteins. In the yeast *Saccharomyces cerevisiae*, the RING finger E3 ligase Ubr1, the recognition component of the N-end rule pathway, cooperates with chaperones to mediate the ubiquitination of misfolded cytosolic proteins for degradation by the proteasome.^50, 51^ The yeast Ub ligase San1 mediates the ubiquitination of misfolded proteins in the nucleus.^52^ With the help of heat-shock protein 70 (Hsp70), San1 also brings excessive cytosolic misfolded proteins to the nucleus for proteasomal degradation.^51, 53^ The yeast HECT Ub ligase Hul5 was recently found to mediate the ubiquitination of misfolded proteins generated by heat shock.^54^ In mammals, the U-box-containing E3 ligase CHIP is known to interact with Hsp70 and promote the delivery of misfolded cytosolic proteins to cellular degradation machinery.^55^ Little is known about the mammalian Ub ligases involved in quality control of misfolded proteins in neurons.

The pathogenesis of many neurodegenerative diseases, including AD, PD, ALS, HD and prion diseases, is associated with and, moreover, at least partly contributed by the downregulation of the UPS.^56, 57^ One major risk factor underlying reduced UPS activities in degenerating brains is aging. Extensive studies have shown that proteasomal activities can gradually decrease with aging, which results in a reduced capacity to degrade misfolded proteins, contributing to the formation of pathological protein aggregates.^27, 28, 29, 31^ Another risk factor is the presence of aggregated proteins that inhibit the activities of UPS components, including the proteasome. For example, aggregated *β*-sheet-rich PrP blocks the opening of the 20S proteasome particle, leading to reduced proteasomal activity.^58^ Ubiquitinated and aggregated tau in AD can block the gate of the 19S catalytic particle by binding to its recognition site, leading to a traffic jam and impaired proteasomal degradation.^30, 32^ In addition, recent studies have shown that aggregates of many other pathogenic proteins in neurodegenerative disorders can directly inhibit proteasome activity.^59, 60, 61, 62^

## The autophagy-lysosome system in neurodegenerative diseases

Autophagy is a process by which cytoplasmic constituents are degraded by the lysosome. Protein quality control via autophagy is particularly important for the timely removal of aggregated forms of pathogenic proteins in neurodegenerative diseases, including tau in AD, *α*-synuclein in PD and polyQ-Htt in HD.^63, 64^ Autophagy can be divided into microautophagy, CMA and macroautophagy, depending on the mechanism by which cellular cargoes are delivered to the lysosome (Figure 1).^65^ Among the three arms of autophagy, the targeted clearance of misfolded proteins is mainly mediated by CMA and macroautophagy. CMA is a selective proteolytic system in which specific misfolded proteins carrying the KFERQ motif are delivered to and degraded in lysosomes. This pentapeptide motif, found in ~30% of cytosolic proteins, is normally buried by protein folding, but it can be exposed on the surface by misfolding or partial unfolding. It is recognized by the chaperone Hsc70 associated with cochaperones.^6^ The substrates are subsequently delivered to the CMA adaptor (lysosomal membrane-associated protein 2A (LAMP-2A) on the lysosomal membrane, unfolded, translocated into the lysosomal lumen and degraded into amino acids. In degenerating neurons, CMA can be constitutively activated to compensate for impaired macroautophagy.^66^

In macroautophagy, a portion of cytoplasmic constituents, such as misfolded proteins and organelles, are segregated by double-membrane structures called autophagosomes and subsequently digested by lysosomal hydrolases (Figure 1). The delivery of misfolded proteins to autophagosomes involves specific adaptors, including the p62/SQSTM-1/sequestosome.^67^ The autophagic adaptor p62 has a UBA (Ub association) domain that interacts with polyubiquitin chains of misfolded proteins and a PB1 domain that mediates self-aggregation to form condensed cargo-p62 complexes.^68, 69, 70^ Cargo-loaded p62 and its aggregated complexes are delivered to autophagic vacuoles through the specific interaction of p62 with light chain 3 II (LC3-II), an active form of LC3, on the surface of autophagic double membrane structures.^71^ By inducing aggregation and eventually delivery to autophagic vacuoles, p62 reduces the toxicity of a free form or oligomeric species of misfolded proteins destined for macroautophagy.^72^ Mutations in the p62 gene have been implicated in the pathogenesis of Paget disease of bone as well as familial and sporadic ALS.^73^ In addition to p62, other autophagic adaptors, such as NBR1, NDP52, optineurin (OPTN), histone deacetylase 6 and NIX26, mediate the delivery of various types of cellular cargoes to autophagic membranes through similar mechanisms.^74, 75^ Once misfolded proteins are loaded to phagophores, the autophagic membrane structures are fused with each other to grow into autophagosomes, which are fused in turn with lysosomes, generating autolysosomes in which cargoes are degraded by lysosomal hydrolases. Autophagosome formation involves a large number of proteins and their post-translational modifications, such as the ATG7-mediated conjugation of ATG5 (autophagy-related protein 5) to ATG12, leading to cleavage and lipidation of LC3-I to form LC3-II (Figure 3).^7, 76, 77^ Upon conversion, cytosolic LC3-II is translocated to autophagic membranes and acts as an anchor to receive cargoes through interaction with autophagic adaptors.

Although misfolded proteins can be immediately and directly delivered to autophagosomes, excess misfolded or damaged proteins and their aggregates that accumulate beyond cellular capacity are temporarily stored in the aggresome, a cytoplasmic inclusion in the microtubule organizing center near the nucleus.^9^ During this process, called aggrephagy, the histone deacetylase 6, in association with molecular chaperones, binds freely floating ubiquitinated aggregates and delivers them via microtubules to a location that minimizes their toxicity until they are finally degraded by the UPS or macroautophagy.^78, 79, 80, 81^ The major components of aggresomes include ubiquitinated proteins as well as specific regulatory proteins involved in the formation and degradation of proteins aggregates, such as p62, ALFY (autophagy-linked FYVE protein) and NBR1 (neighbor of BRCA1 gene).

The functions and survival of neurons heavily depend on the efficient removal of misfolded proteins by autophagy because they cannot dilute cytotoxic proteins by cell division. In addition, autophagy is an intrinsically challenging process in neurons because of their unique cellular structure characterized by the expansion of dendrites and axons. For example, misfolded proteins that have been generated in axons and nerve terminals are packaged on site into autophagosomes and make a long retrograde journey to the cell body, wherein lysosomes are enriched in the perinuclear microtubule-organizing center.^22^ Before reaching the cell body, autophagosomes in the process of retrograde transportation often fuse with late endosomes generated in neurites, resulting in the formation of amphisomes.^23, 24^ This is a time-consuming, difficult and complicated process whose overall efficiency can be adversely affected by many factors, such as aging and genetic mutations. Extensive studies have shown that many components of CMA and macroautophagy are downregulated at the levels of transcription, translation and post-translation as neurons age.^25, 26^ These age-sensitive regulators include the substrate recognizer/carrier Hsc70^82, 83^ and the Hsc70-acceptor LAMP-2A in CMA^84^ as well as Beclin-1 in macroautophagy.^85, 86^ Reduced autophagic activity appears to be pharmaceutically manageable, as the restoration of CMA by maintaining LAMP-2A levels in aging mouse livers has been shown to promote liver health and increase the ability of hepatocytes to degrade damaged proteins.^84^ In addition to reduced autophagic activity in aged neurons, the activities of autophagic components can be adversely affected by interaction with protein aggregates,^87, 88, 89^ which can be excessively generated by age-dependent impairment of the UPS. For example, tau in frontotemporal lobar dementia with Ub-positive inclusions and α-synuclein in PD bind LAMP-2A with an unusually high affinity, leading to a traffic jam during cargo translocation across the lysosomal membrane.^89^ Yet another risk factor underlying dysregulation of autophagy in aged neurons is a genetic mutation in a regulator of autophagy, such as p62, whose mutations are implicated in the pathogenesis of familial and sporadic ALS32, characterized by p62-positive inclusions in affected neurons.^90^

## Protein quality control in AD: Aβ and tau

AD is the most common form of progressive dementia, characterized by cognitive impairment, memory loss and behavioral abnormalities. This protein misfolding disorder is caused by the misfolding and aggregation of amyloid β peptides and tau, which give rise to amyloid plaques and neurofibrillary tangles, respectively.^91^ Aβ is a 42-residue product resulting from two sequential cleavages of the amyloid precursor protein (APP), a transmembrane protein with no clearly defined function. The first cleavage produces a C-terminal fragment, and the fragment is then cleaved by the γ-secretase complex composed of presenilin-1, APH-1, PEN-2 and nicastrin^92^ to generate Aβ, which tends to be misfolded to form aggregates.^10, 11^ Mutations of various genes, including *APP*, can upregulate the production of Aβ, contributing to the pathogenesis of AD.^10, 11^ By contrast, APP and Aβ can be downregulated by the UPS at various steps of processing, from the ER lumen to the plasma membrane.^93^ The first UPS degradation occurs after a nascent APP polypeptide is cotranslationally translocated into the ER lumen, during which its signal peptide is cleaved off. Following translocation, a successfully folded APP mature protein enters the Golgi secretory pathway. However, terminally misfolded APP is degraded via ER-associated degradation in which substrates are unfolded, ubiquitinated, retrotranslocated across the ER membrane and degraded by the proteasome. The targeting by ER-associated degradation involves the E3 Ub ligases HRD1^94^ and Fbxo2.^95^ Proteasomal degradation can also occur when APP arrives at the Golgi apparatus, where APP is ubiquitinated though a K63 linkage by unknown E3 ligases stimulated by ubiquilin-1, leading to the retention of APP without proteasomal degradation.^96^ Even after being presented at the plasma membrane, APP can be internalized to endosomes and enter the endosome-Golgi pathway, where APP can be cleaved to generate Aβ.^97^ The resulting intracellular Aβ is prone to misfolding and is targeted by UPS-dependent protein quality control, which includes the E3 ligase CHIP that mediates the ubiquitination of misfolded proteins for proteasomal degradation.^93^ In contrast to APPs, however, Ub-conjugated Aβ in affected neurons is not properly degraded through the proteasome.^98^

Recent studies have implicated autophagy in the turnover of Aβ. In an AD mouse model overexpressing Aβ, haploinsufficiency of Beclin-1 reduced autophagy and exacerbated AD pathology, as evidenced by A*β* deposition and neurodegeneration, which was rescued by lentiviral administration of Beclin-1.^99^ Conditional mutant mice lacking ATG7 in the central nervous system showed degeneration of pyramidal neurons in the hippocampus and Purkinje cells in the cerebellum.^100^ Genetic inactivation of other autophagic components in neurons, such as ATG5 or ATG17/FIP200, resulted in similar neuronal degeneration.^25, 101^ While the turnover of Aβ involves autophagy, autophagy itself is impaired in the brains of AD patients. For example, affected neurons in AD brains are enriched in autophagosomes and other types of autophagic vacuoles that together act as a major intracellular reservoir of cytotoxic peptides.^102^ The excessive accumulation of immature autophagic vacuoles in senile neurons is associated with increased synthesis of autophagic core components, retrograde transportation of autophagosomes and impaired fusion with lysosomes, contributing to the accumulation of pathogenic Aβ.^103, 104^

**Figure 4:**
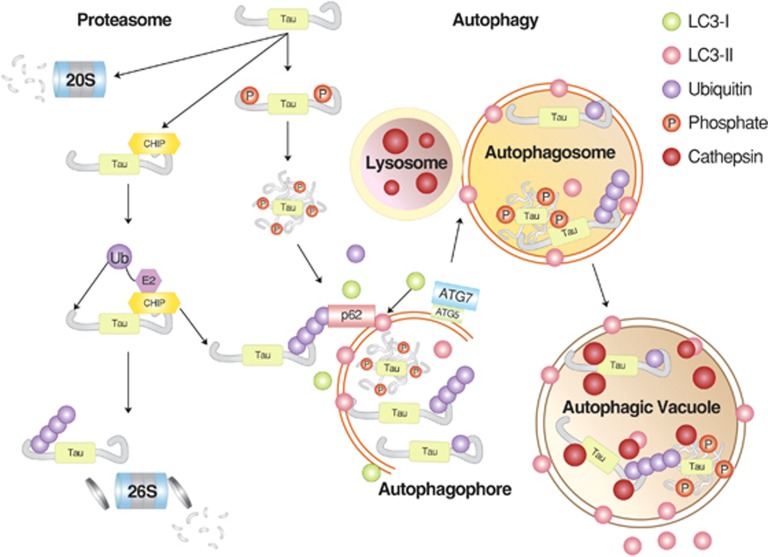
The degradation of tau proteins. Tau can be targeted by both the UPS and macroautophagy, depending on the nature of post-translational modifications that influence folding and solubility. In general, soluble monomeric tau proteins are recognized by molecular chaperones and Ub ligases, such as CHIP, leading to the formation of ubiquitinated tau proteins. It remains unclear as to what extent ubiquitinated tau proteins are actually degraded by the proteasome. Alternatively, the same substrates can be directly delivered to the 20S proteasome without ubiquitination. Some tau proteins prone to rapid aggregation, such as hyperphosphorylated species, can be delivered to p62 and, subsequently, autophagosomes for lysosomal degradation. Modified from Chesser *et al.*^227^

Another hallmark of AD is neurofibrillary tangles composed primarily of phosphorylated tau.^105^ Although neurofibrillary tangles were initially thought to be one of the major causes of AD pathogenesis,^106^ recent studies indicate that a monomeric form of tau with pathological modifications and its soluble oligomers may be more cytotoxic.^12^ The tau protein can lose its function through various proteolytic events, including cleavage by endoproteolytic enzymes such as caspases,^107^ calpain,^108^ aminopeptidases^109^ and thrombin.^110^ However, these cleavages are unlikely to contribute to the clearance of neurofibrillary tangles because the resulting cleavage products with various modifications may aid the development of AD. The first line of defense against tau accumulation is the E3 ligase CHIP, which mediates the ubiquitination of tau (primarily in its phosphorylated form), in collaboration with Hsp70 and Hsp90 (Figure 4).^111^ An *in vitro* study showed that the E2 enzyme Ube2w can also mediate E3-independent ubiquitination of tau.^112^ However, ubiquitinated tau is not a good substrate of the proteasome and thus accumulates as detergent-resistant aggregates, leading to the formation of neurofibrillary tangles in AD. In the process of targeting tau to the proteasome, CHIP also appears to be deposited to neurofibrillary tangles with its substrate and other ubiquitinated proteins.^98, 111^ It has been shown that UPS-dependent clearance of tau is facilitated by overexpressing the molecular chaperone Hsp70, which binds misfolded proteins.^111^ As UPS-dependent degradation of tau is not efficient, autophagy has a close relationship with AD pathogenesis with respect to the formation of amyloid plaques and tau aggregates.^113^ For example, autophagic inhibition by 3-methylamphetamine or cloroquine was shown to slow tau clearance, leading to tau aggregation.^114^ By contrast, rapamycin, an inducer of autophagy, inhibited the accumulation of tau aggregates and neurotocixity using a mouse tau model.^37^ Pharmaceutical inhibition of phospholipase D1, which regulates autophagosome maturation downstream of Vps34, resulted in neuronal accumulation of tau and p62 aggregates.^115^ A subpopulation of caspase-generated tau fragments has been shown to be delivered to autophagic vacuoles.^116^ Defective autophagic flux promotes the formation of tau oligomers and insoluble aggregates. A phosphorylated form of tau shows reduced binding to microtubules and bundling as well as an increased tendency to be found as motile particles.^117^

## Protein quality system in PD: α-synuclein

PD is the most common neurodegenerative movement disorder. It is characterized by decreased motor ability and the loss of dopaminergic neurons in the substantia nigra pars compacta. The major pathogenic agent of PD is a mutant form of α-synuclein, a presynaptic nerve terminal protein.^118^ The activity of mutant α-synuclein as an autosomal dominant cause for PD is associated with point mutations (for example, A53T, A30P and E46K) that render α-synuclein prone to misfolding and aggregation.^119, 120, 121^ The accumulation of aggregated mutant α-synuclein leads to the formation of intracellular inclusions called Lewy bodies (LBs), which serve as the major hallmarks of both sporadic and familial PD. In addition to mutant α-synuclein, LBs contain more than 90 proteins, including PD markers (DJ-1, LRRK2 (leucine-rich repeat kinase 2), Parkin and PINK-1 (PTEN-induced putative kinase 1)) and mitochondria-related proteins, as well as components of the UPS and autophagy, particularly those involved in aggresome formation.^122, 123, 124^ Consistent with the finding that many of the proteins accumulated in LBs are involved in protein quality control, major causative mutations in familial PD are linked to genes in the UPS or autophagic pathways, including α-synuclein, PINK-1, the Ub ligase Parkin, UCH-L1 (Ub carboxy terminal hydrolase L1), DJ-1 (PARK7) and LRRK2/PRAK8.^122^ In the pathogenesis of PD, monomeric and non-fibrillar mutant α-synuclein molecules may be more cytotoxic than fibrillar aggregates, and LBs found in the brains of PD patients may be a consequence of cytoprotective responses.^122, 123, 124^

**Figure 5:**
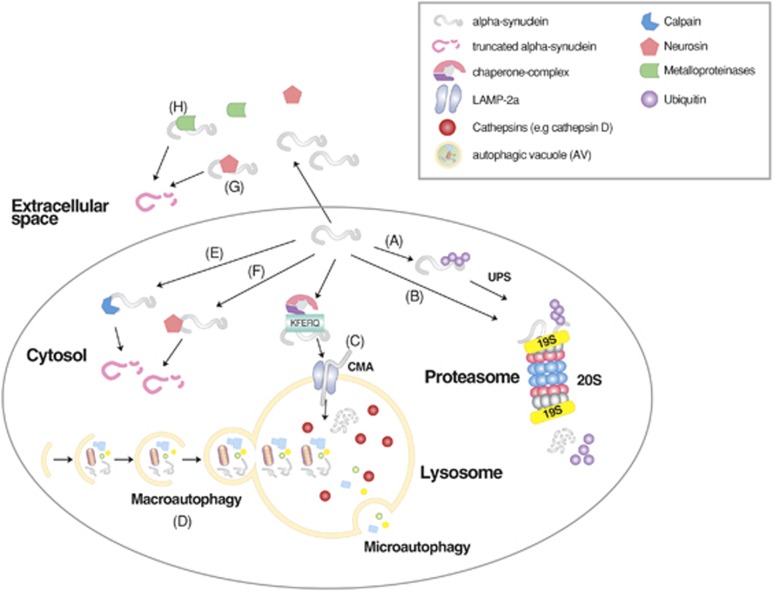
The degradation of α-synuclein by cellular protein quality control. Wild-type and mutant α-synuclein can be targeted by the ubiquitination-dependent UPS (A) and possibly in a manner independent from Ub (B) as well. Monomeric α-synuclein can also be targeted by the CMA (C). By contrast, macroautophagy can degrade monomeric and oligomeric α-synuclein as well as its aggregates (D). Intracellular α-synuclein can also be cleaved by endopeptidases, such as calpains (E) and neurosin (F). Extracellular α-synuclein can be cleaved by neurosin (G) and metalloproteinases (H). The resulting proteolytic cleavage products are thought to contribute to the cytotoxicity of α-synuclein. Modified from Xilouri *et al.*^228^

Wild-type α-synuclein has been shown to be ubiquitinated and degraded by the proteasome using *in vitro* assays^89, 125, 126^ and cultured neuronal cells under proteasomal inhibition.^127, 128, 129^ However, other studies have suggested that ubiquitination is not needed for proteasomal degradation of α-synuclein (Figure 5).^130, 131^ Proteasomal degradation of α-synuclein has been shown to be facilitated by its phosphorylation at Ser129.^132^ Several regulatory proteins of the UPS were implicated in the turnover of soluble α-synuclein in the cytosol, including Ub ligases CHIP,^133^ SIAH,^134, 135^ MDM2^136^ and HRD1.^137^ A subpopulation of α-synuclein associated with membranes in the endosome-lysosome pathway has been shown to be targeted by the Ub ligase Nedd4.^138^ In addition to Ub ligases, rare mutations in the deubiquitinating enzyme UCH-L1 have been associated with familial, early onset PD.^139^ PD-linked mutants of UCH-L1 contain only partial deubiquitinating activities, contributing to the accumulation of α-synuclein in presynaptic terminals.^140^ The role of UCH-L1 in PD pathogenesis is in part attributed to its activity as an E3 ligase, whereby it mediates K63-linked ubiquitination in its dimer form.^141^ The overall importance of the UPS in the turnover of α-synuclein is further supported by the finding that conditional knockout mice lacking Psmc1, a proteasomal subunit, in nigral or forebrain neurons resulted in the formation of intraneuronal LB-like inclusions positive for Ub and α-synuclein associated with neurodegeneration.^142^ While soluble α-synuclein is degraded by the proteasome, its filamentous form can interact directly with the 20S core of the proteasome and decrease its proteolytic activity.^61^ Consistently, proteasome misregulation has been observed in the substantia nigra of PD patients.^143^

Recent studies have shown that α-synuclein can be degraded by CMA through a specific CMA recognition motif.^89, 144^ However, the A30P and A53T PD-linked mutants have unusually high affinity for the CMA adaptor LAMP-2A and are not efficiently delivered to the lysosomal lumen, resulting in a traffic jam in CMA.^89, 145^ This, in turn, can trigger compensating macroautophagy.^146^ The hydrolysis of CMA-targeted α-synuclein in the lysosomal lumen involves cathepsin D, a primary lysosomal protease.^147, 148^ Although α-synuclein in a monomeric or soluble oligomeric form can be targeted by both the UPS and the CMA, its aggregates are directed to the lysosome via macroautophagy. The role of macroautophagy in α-synuclein degradation was suggested by the finding that α-synuclein is accumulated in the lysosome of cultured neuronal cells under macroautophagic inhibition, whereas the lysosomal targeting of PD-linked mutant α-synuclein was attenuated under the same conditions.^33, 149^ Pharmacological activation of macroautophagy using rapamycin, a mammalian target of rapamycin (mTOR) inhibitor, facilitated the degradation of both wild-type and mutant α-synuclein.^138, 150^ The clearance of α-synuclein by macroautophagy was further shown in transgenic mice virally overexpressing Beclin-1, an autophagic regulator.^150^

## Protein quality control in HD: mutant huntingtin proteins

HD is an autosomal dominant neurodegenerative disorder that affects ~5–10 individuals per 100 000.^151^ Affected individuals suffer from progressive motor and cognitive declines associated with loss of self and spatial awareness, depression, dementia and increased anxiety. This progressive neurodegenerative disease is caused by the aggregation of mutant huntingtin (mHTT) proteins. The wild-type huntingtin protein (HTT) contains a stretch of the glutamine residue, called polyQ tract, which is encoded by a repeat of the codon CAG within exon 1 of the *HTT* gene.^152, 153^ The length of the CAG repeat varies between individuals and generations, ranging on average between 16 and 20 repeats.^154^ In affected individuals, the CAG repeat expands to >35 in number, giving rise to the elongated polyQ tract of mHTT proteins that are prone to aggregation and toxic to neurons.^14, 155^ PolyQ inclusions are abundant in highly ordered amyloid fibers with enriched β-sheets and low detergent solubility.^156^ PolyQ inclusions may be a consequence of a protective mechanism to sequester small oligomeric forms of mHTT, which are highly cytotoxic to neurons.^157^ Extracellular polyQ aggregates can be internalized by cells to initiate a new round of polyQ aggregation, suggesting that mHTT may act as an infectious agent through a mechanism observed in prion diseases.^158^

Despite the importance of mHTT in the pathogenesis of HD, surprisingly little is known about the mechanism by which cytotoxic mHTT is removed from the cell. This is perhaps because mHTT is a poor substrate for all known proteolytic pathways, including UPS, CMA, and macroautophagy. Moreover, extensive studies have shown that mHTT acts as an inhibitor of proteolytic machineries, often in the process of its turnover.^159^ For example, mHTT inclusions in the brains of HD patients and HD mice are enriched in the components of the UPS, such as Ub and ubiquitinated HTT, because mHTT species can be initially tagged with Ub but are poor substrates for the proteasome.^160^ It has been suggested that the accumulation of mHTT inclusions is not a consequence of direct proteasomal inhibition but rather result from the gross failure of protein quality control systems in association with the sequestration of molecular chaperones.^161^

Wild-type HTT can be degraded by CMA,^162^ during which Hsc70 recognizes two KFERQ-like motifs, KDRVN at residues 99–103 and NEIKV at residues 248–252.^159^ Like HTT, mHTT can also be recognized by Hsc70 for CMA degradation.^159^ However, the polyQ expansion of mHTT delays the delivery of mHTT across the lysosomal membrane because mHTT has a higher affinity for Hsc70 and LAMP-2A.^159^ Failure to promptly deliver the initially targeted mHTT to the lysosome results in a traffic jam in CMA-dependent autophagic degradation, leading to a secondary side effect in proteostasis. Failure to degrade mHTT results in the accumulation of perinuclear cytoplasmic aggregates and intranuclear inclusions in the neurons of patients with HD.^162^

Core components of macroautophagy, such as LC3, are typically upregulated in various HD mouse models and in neuronal and non-neuronal cells in patients with HD.^163, 164^ The apparent upregulation of macroautophagy is associated with the excessive formation of cargo-free autophagic vacuoles, possibly because the delivery of cargoes to autophagic vacuoles is impaired.^163^ As the autophagic flux is reduced, components of macroautophagy, such as p62, LC3-II, mTOR and Beclin-1, were found to be deposited in the striatum of HD transgenic mice.^165^ The sequestration of autophagic regulators in mHTT inclusions, such as mTOR, contributes to the increased synthesis of autophagic core components.^85, 166^ Thus, HD disease progression is exacerbated by reduced activities of macroautophagy associated with HTT inhibition of macroautophagy in an age-dependent manner.

## Protein quality control in prion diseases: scrapie prion protein

Prion diseases, also known as transmissible spongiform encephalopathies, are infectious neurodegenerative disorders in humans and animals that affect the brain and nervous system, leading to spongiform vacuolation and severe neuronal loss.^167^ Prion diseases in animals include nature scrapie in sheep and goat,^168^ bovine spongiform encephalopathy (also known as mad cow disease) in cattle,^169^ chronic wasting disease in elk and deer,^170^ and feline spongiform encephalopathy in domestic cats.^171^ In humans, these fatal protein misfolding disorders include kuru^172^, Creutzfeldt–Jakob disease^173^, Gerstmann–Sträussler–Scheinker syndrome^174^, fatal familial insomnia^175^ and new variant CJD (a human equivalent to bovine spongiform encephalopathy/mad cow disease).^167^

The transmissible agent common to these transmissible diseases is scrapie prion protein (PrP^Sc^), an abnormally misfolded isoform of the host-encoded cellular prion protein (PrP^C^).^18^ PrP^C^ is a glycosylphosphatidyl inositol-linked glycoprotein enriched in α-helical structure. This cell surface protein with no clearly defined function is initially translated as a 253-residue polypeptide and enters the ER wherein its signal peptide is cleaved off, generating a 208-residue mature protein. The mature PrP^C^ polypeptide undergoes folding and is conjugated with sugar moieties during transportation via the Golgi-secretory pathway. In this process, a soluble form of misfolded PrP^C^ is normally degraded by various protein quality control systems, including Ub-dependent ER-associated degradation. Compared with PrP^C^, however, PrP^Sc^ is enriched in β-sheets and tends to form aggregates that are at least partially resistant to all known cellular protein quality control systems.^17, 18, 19^ Moreover, PrP^Sc^ can interact with PrP^C^ and facilitate the conversion of PrP^C^ into PrP^Sc^, which, in turn, can convert more PrP^C^ into PrP^Sc^, resulting in the accumulation of misfolded and aggregated PrP^Sc^ in the brain.^176, 177, 178^ Through this seeding-nucleation process, a small quantity of invading PrP^Sc^ is enough to trigger the autocatalytic conversion of host PrP^C^ into PrP^Sc^.^179, 180^ The transmissible nature of PrP^Sc^ has been demonstrated by the finding that the inoculation of small quantities of PrPSc into animals led to characteristics of prion diseases.^177, 178^

Compared with the clinical importance of PrP^Sc^, surprisingly little is known of its turnover. In principle, as the conversion of PrP^C^ into PrP^Sc^ requires significant refolding and conformational changes in folding, this process may involve chaperones and Ub ligases of UPS-dependent protein quality control. Indeed, recent studies in *S. cerevisiae* suggest that Hsp70, Hsp40 and Hsp26 may loosen prion fibrils, whereas Hsp104 fully disassembles the fibrils into shorter fragments.^181^ In mammalian cells, the chaperones GroEL and Hsp104 were shown to facilitate the conversion of PrP^C^ into PrP^Sc^ in the presence of a small amount of PrP^Sc^ that served as a seed.^182^ In addition, Hsc70, a recognition component of CMA, was shown to bind to PrP^C^.^183^ Despite the implication of chaperones in the turnover of PrP^C^, it appears that PrP^Sc^ is not a good substrate of the UPS. Moreover, recent studies have shown that PrP^Sc^ binds to the 20S proteasome without further processing and thus blocks substrate entry into the proteolytic chamber, leading to proteasomal failure.^62, 184^ PrP^Sc^ may also bind to the external surface of the 20S particle and induce an allosteric stabilization of the closed state of the 20S proteasome.^58, 185^ Consistent with these findings, prion diseases are associated with impaired activities of the UPS.^185^ As a consequence of proteasomal inhibition, cellular Ub conjugates are excessively accumulated in mouse brain infected with ME7 scrapie train.^185^

Prion diseases are associated with misregulation of autophagy as evidenced by the formation of giant autophagic vacuoles in experimental scrapie in hamsters.^186^ These autophagic vacuoles often grow in size and number as neurons age, eventually occupying the entire volume of the affected neurites.^187^ The formation of giant autophagic vacuoles is caused by the reduced flux of autophagy in combination with endosomal/lysosomal dysfunction, which may contribute to the pathogenesis of prion diseases.^187^ Although a study showed that recombinant PrP^C^ mutants (V203I, E211Q and Q212P) overexpressed in neuroblastoma cells were converted to PrP^Sc^-like aggregates and delivered to aggresomes,^188^ there is no evidence that PrP^Sc^ is efficiently processed by autophagic pathways. Instead, recent studies indicate that prion proteins adversely affect autophagy, as exemplified by the finding that the overexpression of a PrP^C^-like protein, Doppel (Dpl), in neurons resulted in the progressive death of Purkinje cells in prion-lacking Ngsk mice.^189^ As further described in the following sections, one way to facilitate the clearance of PrP^Sc^ is to use small molecules that stimulate autophagy.^35, 190, 191^

## Protein quality control in ALS: SOD and TDP-43

ALS is a progressive paralytic disease characterized by selective degeneration and death of motor neurons associated with the accumulation of misfolded proteins and insoluble inclusions.^20^ Although indistinguishable in clinical symptoms, this protein misfolding disorder can be divided into sporadic ALS, which accounts for ~82% of all ALS cases, and familial ALS.^20^ Mutations in ALS may occur in genes encoding key components of protein quality control. This group of mutant ALS proteins includes dynein and dynactin, both involved in the retrograde transport of autophagosomes from axons to the cell body,^192, 193^ the autophagic adaptor p62,^73^ and the UBA-containing proteins Ubqln2 and Optineurin.^194^ Another group of ALS mutations generates proteins with abnormal folding, leading to aggregation and the formation of insoluble inclusions.^20^ This latter group includes SOD1, TDP-43, and FUS/TLS (Fused in Sarcoma/Translocated in Sarcoma).^20, 195^ Approximately 20% of familial ALS cases are caused by over 140 different point mutations of SOD1, a soluble cytosolic enzyme that dismutates superoxide radicals to H_2_O_2_.^196^ SOD1 mutants are mostly dominant and causative to the death of affected motor neurons because they tend to be misfolded and form protease-resistant aggregates.^195^ Another ASL-relevant gene is TDP-43, in which mutations account for ~5% of sporadic ALS and 3% of familial ALS cases.^20^ This hnRNP family member can bind to RNA in a single-stranded and sequence-specific manner, which is required for many RNA processes.^197^ One unique aspect of TDP-43 is the property of its C-terminal tail to be prone to misfolding and aggregation.^197, 198^ Like other pathogenic mutant proteins in neurodegenerative diseases, misfolded SOD1 and TDP-43 mutants are initially targeted for degradation by the components of the UPS, such as chaperones and Ub ligases.^20^ Owing to their tendency to aggregate, however, the targeted mutants escape during the delivery process to the proteasome, some of which are redirected to autophagy. ALS mutants resistant to the UPS and autophagy are aggregated together to form intracellular inclusions containing Ub and Ub ligases found in familial ALS mutant mice^199, 200^ and post-mortem spinal cord of sporadic ALS patients.^201, 202, 203^ It was reported that the insoluble inclusions typically become visible in the brain stem and spinal cord at the onset of ALS symptoms and progressively accumulate throughout late stages.^204^ Although large inclusions are clinical hallmarks of ALS symptoms, they are unlikely to be toxic to neurons. They may, however, be a neuroprotective phenomenon, as it was suggested that monomeric and oligomeric misfolded ALS proteins are the actual toxic substance in motor neurons.^195^

Autophagy is often misregulated in the spinal cord of sporadic ALS patients, as evidenced by the excessive formation of autophagosomes.^205^ The autophagic misregulation can be partially explained by findings stating that inclusions observed in ALS patients can impair protein quality controls by sequestering various components ranging from proteasomal subunits and Ub ligases, such as Dorfin, to molecular chaperones HSP70 and HSP40 and the motor protein dynein involved in cargo delivery to the aggresome.^5, 206, 207^ Monomeric or oligomeric ALS proteins can also directly inhibit both proteasomal activity^197, 198, 208, 209^ and autophagic flux.^210, 211, 212^ Moreover, it has been shown that reduced proteasomal activity can promote the accumulation of ALS protein aggregates.^213^ Thus, one mechanism underlying the pathogenesis of ALS is a vicious cycle between misfolded proteins and proteolytic pathways, which accelerates the excessive accumulation of insoluble inclusions, leading to the death of affected motor neurons.

## Targeting autophagy for therapy of neurodegenerative diseases

Substantial benefits of therapy could be achieved with agents that promote the degradation of pathogenic proteins underlying neurodegenerative diseases. Many small molecules that induce autophagy have been developed and shown to be effective in removing pathogenic proteins. The therapeutic activities of the autophagic inducer rapamycin, an inhibitor of mTOR, have been demonstrated using transgenic mouse models of neurodegenerative diseases, such as AD mice expressing mutant APP,^36, 38^ AD mice expressing tau,^37^ HD mice expressing mHTT,^214^ PD mice expressing mutant α-synuclein^33^ and prion disease mice expressing PrP^Sc^.^35^ The overall results indicate that rapamycin promotes the clearance of these pathogenic protein aggregates, improves cognition and behavior and ameliorates neuropathology and neurodegeneration in the brains of these transgenic mouse models. Similar therapeutic benefits were obtained using analogs of rapamycin, such as CCI-779, which was shown to reduce mHTT aggregates, leading to improved motor behaviors in HD transgenic mice.^215^ In contrast, rapamycin worsened autophagic functions and neuron degeneration in a SOD1(G93A) transgenic mouse model of ALS^212^ and 1-methyl-4-phenyl-1,2,3,6-tetrahydropyridine (MPTP) neurotoxin models of PD,^216^ suggesting that autophagic induction may exert adverse effects on certain neurodegenerative conditions.

Various autophagic inducers were exploited to enhance the clearance of pathogenic protein aggregates in neurodegenerative diseases by targeting the ULK1 kinase AMP-activated protein kinase (AMPK) or cAMP–inositol 1,4,5-trisphosphate.^13^ An mTOR-independent macroautophagy inducer, Rilmenidine, was shown to improve motor ability and the clearance of mHTT fragment in transgenic HD mice.^217^ The mood stabilizer lithium, known to inhibit inositol monophosphatase and the phosphoinositol cycle, promoted the degradation of various protein aggregates including PrP^Sc^ of prion disease,^191^ mHTT of HD, α-synuclein of PD^218^ and SOD1 G93A of ALS.^219, 220^ Trehalose is a natural disaccharide product with pharmacological chaperone activity that exerts a protective role against various environmental stresses.^221^ This mTOR-independent autophagy activator was shown to enhance the clearance of mHTT in cultured cells, reduce the toxicity of mHTT and improve motor ability and lifespan in transgenic HD mice.^221, 222^ Trehalose promoted the clearance of A30P and A53T α-synuclein mutants in cultured PD model cells.^221^ The natural flavone finsetin and related compounds that activate autophagy through both target of rapamycin complex 1 (TORC1) and AMPK activities showed protective effects in neurodegenerative models.^223^ Protein phosphatase 2A agonists that inhibit tau hyperphosphorylation and activate autophagy through TORC1 and AMPK are under clinical trials for AD.^224^ Not surprisingly, a synergistic effect was obtained when rapamycin and Trehalose were combined to remove pathogenic protein aggregates of HD and PD.^221^ The combination of rapamycin and the IMPase inhibitor lithium was also shown to reduce the toxicity of mHTT.^34^ These results suggest that the combination therapy based on an mTOR inhibitor and an mTOR-independent activator may need to be further exploited for therapeutic application, although off-target effects are expected to increase. Collectively, these studies demonstrated that autophagic inducers have potential as therapeutic agents for selected neurodegenerative diseases. The overall effects of these reagents on a broad range of biological processes in neurons and non-neuronal cells require further investigation.

## Concluding remarks

It is estimated that there will be two billion people over the age of 60 by 2050. One common biochemical mechanism underlying most neurodegenerative disorders is the failure of protein quality control to degrade or remove misfolded proteins in the brains of aged persons. The disease-causing misfolded proteins are generated over the course of aging by post-translational modifications (for example, endoproteolytic cleaves and phosphorylation) of native proteins (for example, amyloid β and tau in AD) or genetic mutations of otherwise non-pathogenic proteins (for example, HTT in HD, α-synuclein in PD, PrP^C^ in prion disease and SOD1 and TDP-43 in ALS). These pathogenic agents tend to aggregate into oligomers with enriched β-sheet content, which can further grow into fibrillar inclusion bodies or extracellular plaques, serving as clinical hallmarks of many neurodegenerative diseases. β-Sheet-enriched aggregates can impair—either directly or indirectly—the UPS as well as CMA and macroautophagy by interacting with various cellular molecules, including key components of proteolytic pathways. This results in the reduced ability of protein quality control, which further accelerates the accumulation of cytotoxic aggregates. This exacerbating cycle between misfolded proteins and protein quality control is particularly toxic to aged neurons as the ability of these post-mitotic cells to cope with such difficulties is naturally reduced over the course of aging. As a consequence of these unfortunate events, neurodegenerative diseases are typically associated with global failures of all proteolytic pathways.

A significant portion of cellular proteins is misfolded during translation/folding or while functioning as folded proteins, either spontaneously or under cellular stresses. Most abnormally folded cellular proteins in the human proteome can be efficiently removed through the cooperative work of the UPS, CMA and macroautophagy. In contrast, the aforementioned pathogenic proteins are commonly resistant to those proteolytic pathways, perhaps because their β-sheet-enriched folds are difficult for molecular chaperones to loosen up. These substrates, without being fully unfolded, cannot be properly fed into the proteasomal cylinder, may be stuck within the narrow cylinder of the proteasome, or may not readily dissociate from the components (for example, LAMP-2) of CMA while being delivered across the lysosomal membrane. One strategy to enhance the clearance of pathogenic proteins is to enhance the activities or levels of molecular chaperones engaged in the UPS, as demonstrated by a study in which the overexpression of the molecular chaperone Hsp70 accelerated the proteasomal degradation of tau.^111^ Another strategy is to activate the molecular chaperones (for example, Hsc70), carriers (for example, histone deacetylase 6) and/or adaptors (for example, LAMP-2) of CMA, as a few studies have shown that the augmentation of CMA enhanced the removal of pathogenic misfolded proteins.^8, 159, 225^ One common limitation of the UPS and CMA is that substrates should be at least partially or completely unfolded into nascent polypeptides before they are fed into the proteasome or lysosome. By contrast, the degradation by macroautophagy does not involve an ATP-dependent unfolding step, making this lysosomal proteolysis an ideal quality control system for aggregation-prone misfolded proteins. In addition, although autophagic flux is often reduced in affected neurons in most neurodegenerative diseases, the functions of core autophagic machinery appear to remain largely intact, as several studies have shown that the alteration of autophagic regulators such as mTOR fully restored the autophagic flux. As such, many small molecule compounds were developed to induce macroautophagy and demonstrated to enhance the clearance of cytotoxic protein aggregates. As the mTOR pathway is emerging as a promising drug target, known mTOR-dependent autophagic inducers were successfully used to enhance the clearance of various pathogenic protein aggregates, improve cognition and behavior, and ameliorate neurodegeneration in the brains of various transgenic mouse models. Other regulators of autophagy, such as the ULK1 kinase AMPK, are also being actively exploited as potential drug targets, with synergistic effects between rapamycin and an mTOR-independent autophagic inducer. Although it is increasingly clear that autophagy inducers have therapeutic potential to remove protein aggregates, it should be noted that most of these studies use transgenic mice overexpressing pathogenic proteins that have already formed high levels of insoluble inclusions. The activities of these compounds on a broad range of biological processes, including off-target effects, should be further investigated under more physiologically relevant conditions.
